# Platelets Rich Plasma Increases Antioxidant Defenses of Tenocytes via Nrf2 Signal Pathway

**DOI:** 10.3390/ijms241713299

**Published:** 2023-08-27

**Authors:** Alessia Tognoloni, Desiree Bartolini, Marco Pepe, Antonio Di Meo, Ilaria Porcellato, Kubra Guidoni, Francesco Galli, Elisabetta Chiaradia

**Affiliations:** 1 Department of Veterinary Medicine, University of Perugia, 06126 Perugia, Italy; alessia.tognoloni@studenti.unipg.it (A.T.); marco.pepe@unipg.it (M.P.); antonio.dimeo@unipg.it (A.D.M.); ilariaporcellatodvm@gmail.com (I.P.); kubra.demiralay@studenti.unipg.it (K.G.); 2 Department of Pharmaceutical Sciences, University of Perugia, 06122 Perugia, Italy; desiree.bartolini@unipg.it (D.B.); francesco.galli@unipg.it (F.G.)

**Keywords:** tenocytes, platelet-rich plasma, Nrf2, protein oxidation, tendinopathy, oxidative stress

## Abstract

Tendinopathies are common disabling conditions in equine and human athletes. The etiology is still unclear, although reactive oxygen species (ROS) and oxidative stress (OS) seem to play a crucial role. In addition, OS has been implicated in the failure of tendon lesion repair. Platelet-rich plasma (PRP) is rich in growth factors that promote tissue regeneration. This is a promising therapeutic approach in tendon injury. Moreover, growing evidence has been attributed to PRP antioxidant effects that can sustain tissue healing. In this study, the potential antioxidant effects of PRP in tenocytes exposed to oxidative stress were investigated. The results demonstrated that PRP reduces protein and lipid oxidative damage and protects tenocytes from OS-induced cell death. The results also showed that PRP was able to increase nuclear levels of redox-dependent transcription factor Nrf2 and to induce some antioxidant/phase II detoxifying enzymes (superoxide dismutase 2, catalase, heme oxygenase 1, NAD(P)H oxidoreductase quinone-1, glutamate cysteine ligase catalytic subunit and glutathione, S-transferase). Moreover, PRP also increased the enzymatic activity of catalase and glutathione S-transferase. In conclusion, this study suggests that PRP could activate various cellular signaling pathways, including the Nrf2 pathway, for the restoration of tenocyte homeostasis and to promote tendon regeneration and repair following tendon injuries.

## 1. Introduction

Tendinopathies are disabling disorders that affect 30–50% of human and equine athletes [1,2,3]. Overall, tendon injuries are often the result of both impairments of cellular functions and extracellular matrix (ECM) alterations [4], mainly caused by the molecular factors released during exercise or/and related to excessive mechanical loading. Moreover, tendon tissue has a limited healing capacity that has been attributed to low cell density and a low supply of oxygen and nutrients [5,6]. After injuries, tendon homeostasis is seldom restored, and tendons often fail to regain full function. Reparative scar tissue has a different histological and molecular composition than healthy tendon tissue, with dense collagen fibers that confer inferior mechanical properties, which increases the risk of secondary injuries [4,7].

There is growing evidence that suggests the involvement of oxidative stress (OS) in the onset and progression of tendon degeneration and failed healing response [8,9,10,11,12]. Mitochondrial generation of reactive oxygen species (ROS) in muscles during physical activity and exercise-induced hyperthermia are the major sources of ROS in tendons. Moreover, following tendon injuries, factors including hypoxia–reoxygenation consequent to the ischemia/reperfusion and the release of inflammatory cytokines and nitric oxide in tendon lesions can trigger OS in tendon tissues [13,14,15,16].

Contrastingly, antioxidants can reduce ROS overload, alleviate inflammation in injured tendons and can contribute to tendon repair [17,18,19]. The adaptative cellular antioxidant response is mainly orchestrated through the activation of erythroid nuclear factor 2-related factor 2 (Nrf2) pathway. This transcription factor modulates the expression of antioxidant enzymes and stress response proteins, which play a fundamental role in maintaining cellular redox balance and cell homeostasis [20,21]. Recent evidence suggests that Nrf2 activation accelerates the wound healing process and promotes the repair of some tissues (skin). However, to date, little is known about the role of Nrf2 in the tendon healing process, even if it seems to drive the effects of regenerative therapies, including platelet-rich plasma (PRP) [22,23,24]. This autologous preparation has proven to have high regenerative potential for healing various tissues such as tendon, ligaments, muscles and cartilage. The trophic molecules and growth factors (GFs) contained in PRP seem to stimulate tendon tissue repair by increasing the synthesis of collagen and ECM proteins and by promoting tenocyte proliferation and migration [25,26,27,28,29].

In order to gain a better understanding of the various clinical outcomes of PRP therapy under OS conditions and broaden our knowledge of safe PRP clinical applications, this in vitro study investigates the effects of PRP in tenocytes exposed to hydrogen peroxide (H_2_O_2_)-induced OS and the Nrf2-mediated antioxidant response. To do this, equine tendon cells obtained from the equine superficial digital flexor tendon (SDFT), which is functionally equivalent to the human Achilles tendon [30], were used. The results may help to clarify the molecular mechanism underlying the regenerative properties of PRP, providing new knowledge about the use of these blood-derived products in tendon healing.

## 2. Results

### 2.1. Phenotypical Characterization of Tenocytes Cell Culture

**Figure 1:**
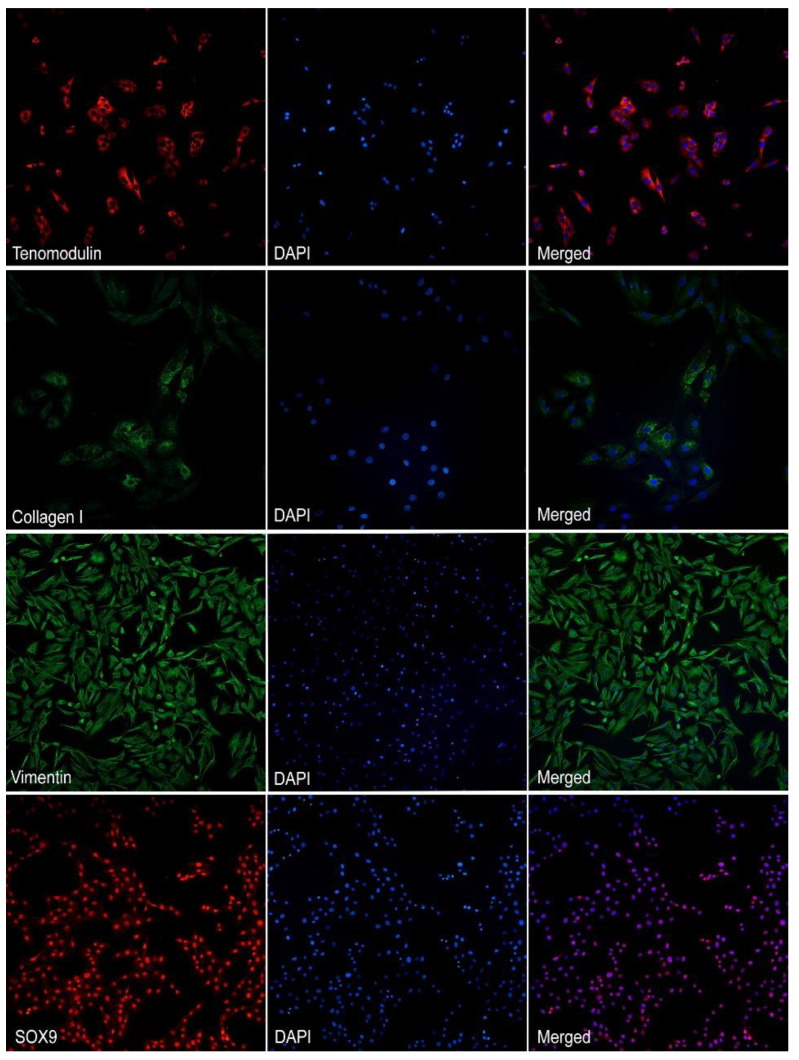
Fluorescent images of tenocytes immunolabeled with tenomodulin (red) (200× magnification), collagen 1 (green) (400× magnification), vimentin (green) (200× magnification), and SOX9 (red) (200× magnification). Nuclear counterstaining was performed with DAPI (blue).

The primary cell cultures of equine tenocytes were checked by using specific antibodies against protein markers, before using cells for the experiments, in order to confirm the tenocyte phenotype. Results showed a strong and finely fibrillar cytoplasmic fluorescence for tenomodulin; similar results were obtained with vimentin. The expression of SOX9 was moderate and localized to the nuclei of the cultured cells. Collagen I was observed as an irregular and finely granular cytoplasmic positivity, variably expressed in the cultured tenocytes (Figure 1).

### 2.2. PRP Protects Tenocytes Exposed to H_2_O_2_

**Figure 2:**
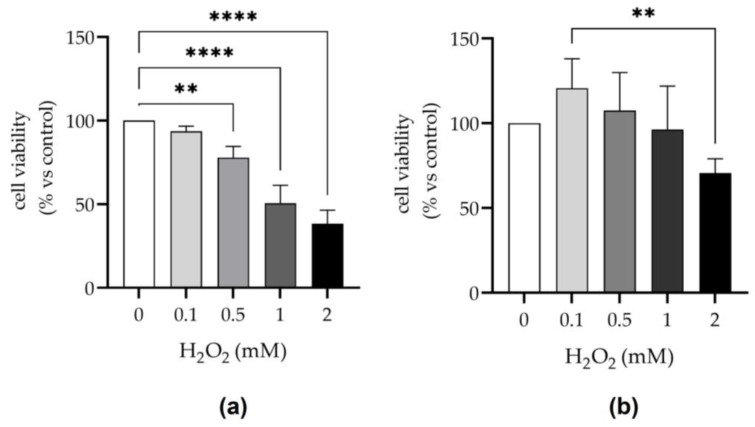
Effect of H_2_O_2_ on tenocyte viability in medium supplemented with (**a**) 10% FBS or (**b**) 10% PRP. Cells were treated with different concentrations of H_2_O_2_ (0, 0.1, 0.5, 1, 2 mM) for 24 h. Cell viability was measured via MTT assay. Data of four independent experiments performed in triplicate are expressed as the mean ± SD. Cell viability was calculated as percentage of ratio between the OD (optical density) of the samples treated with H_2_O_2_ and OD of the respective control. ** *p* < 0.01. **** *p* < 0.0001.

To determine the effects of H_2_O_2_ and PRP on cell viability, tenocytes were exposed to increasing doses of H_2_O_2_ (0, 0.1, 0.5, 1, 2 mM) in medium supplemented with 10% fetal bovine serum (FBS) or 10% PRP for 24 h. As shown in Figure 2, H_2_O_2_ induces a significant dose-dependent decrease in cell viability of tenocytes cultivated in medium supplemented with FBS (*p* < 0.01; *p* < 0.0001). Otherwise, the replacement of FBS with PRP protected cells from H_2_O_2_ effects. In this condition, cells exposed to 0.1–1 mM of H_2_O_2_ showed a cell viability similar to or higher than those observed in the cell control. The lowest cell viability was observed in PRP medium only at very high H_2_O_2_ concentrations, i.e., 2 mM, compared to controls, even if not statistically significant. Remarkably, the same concentration of H_2_O_2_ (2 mM) induced an approximately 60% decrease in cell viability in medium with FBS.

### 2.3. PRP Reduces H_2_O_2_-Induced Protein Oxidation

**Figure 3:**
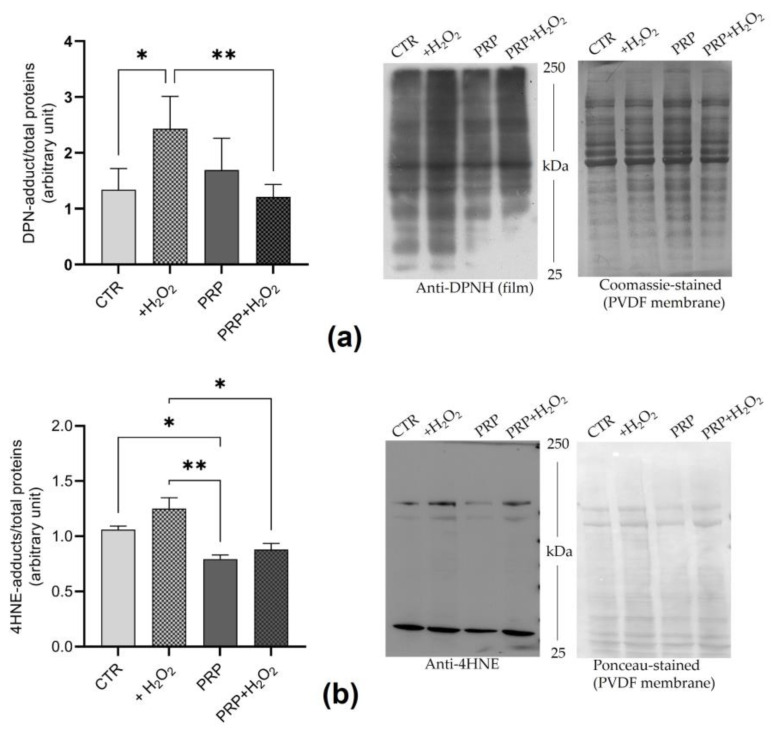
Protective effect of PRP on H_2_O_2_-induced protein oxidation. Cells were treated with/without H_2_O_2_ (1 mM) for 24 h in medium with FBS (10%) (CTR) or PRP (10%). (**a**) Carbonylated protein detected as DNP-protein adducts: Bar graph and representative image of immunoblotting and relative blue Coomassie-stained PVDF membrane used for data normalization. Data obtained from three independent experiments were reported as means ± SD. * *p* < 0.05, ** *p* < 0.01. (**b**) 4-HNE- proteins: bar graph and representative image of immunoblotting and relative blu Comassie-stained PVDF membrane used for data normalization. Data obtained from three independent experiments were reported as means ± SD. * *p* < 0.05, ** *p* < 0.01.

To test the antioxidant effects of PRP, tenocytes were exposed to H_2_O_2_ (1 mM) in medium with FBS (CTR) or PRP (10%) for 24 h, and the oxidative damage of proteins was evaluated. This concentration of H_2_O_2_ was chosen based on previous results, as it induced significant cell death that is reduced by PRP. In particular, the levels of protein carbonylation as 2,4-dinitrophenylhydrazine (DNP)-protein adducts and the levels of 4-Hydroxynonenal (4-HNE)-protein adducts were measured. A significant increase (*p* < 0.05) in protein carbonylation was observed in the tenocytes exposed to H_2_O_2_ in medium supplemented with FBS with respect to the controls (Figure 3a). Moreover, no significant changes were observed by comparing the levels of carbonylated protein in control cells and tenocytes exposed in PRP medium with or without H_2_O_2_ (Figure 3a). The results proved that PRP counteracts the carbonylation of proteins as the levels of DNP-protein adducts treated in cells with H_2_O_2_ in PRP medium are significantly lower than those treated with FBS medium (Figure 3b).

Similarly, the PRP was able to significantly reduce (*p* < 0.05) the levels of 4-HNE-proteins. Tenocytes exposed to H_2_O_2_ in FBS medium showed lower levels of 4-HNE-proteins compared to cells in PRP medium with or without H_2_O_2_ (*p* < 0.05).

### 2.4. PRP Induces Nuclear Translocation of Nrf2

**Figure 4:**
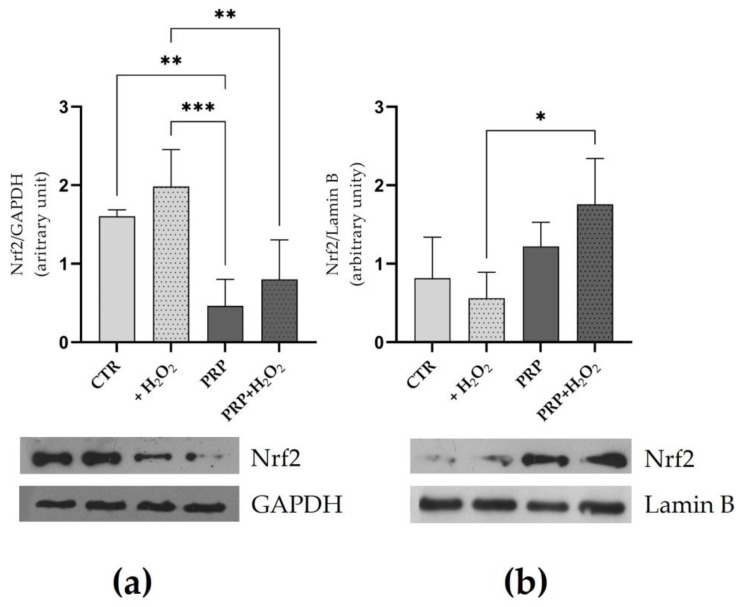
PRP effect on cytosolic (**a**) and nuclear (**b**) levels of Nrf2. Cells treated with or without H_2_O_2_ (1 mM) for 24 h in medium with FBS (10%) (CTR) or PRP (10%) were lysed to extract cytoplasmic and nuclear proteins. The protein levels Nrf2 in both fractions were evaluated via Western blot analysis. Glyceraldehyde-3-phosphate dehydrogenase (GAPDH) and Lamin B were used as cytosolic and nuclear housekeeping proteins, respectively. Results are presented as means ± SD of three separated experiments. * *p* < 0.05; ** *p* < 0.01, *** *p* < 0.001.

To investigate the putative effect of PRP on the activation of Nrf2 signaling in tenocytes, the cytosolic and nuclear levels of this transcription factor were analyzed. As shown in Figure 4, PRP can induce Nrf2 nuclear translocation in pro-oxidizing conditions. In fact, PRP significantly reduced the cytosolic levels of Nrf2 in tenocytes either in the absence or in the presence of H_2_O_2_. On the contrary, the nuclear levels of Nrf2 increased in cells exposed to the H_2_O_2_ in medium with PRP.

### 2.5. PRP Modulates Levels and Activity of Antioxidant Enzymes

**Figure 5:**
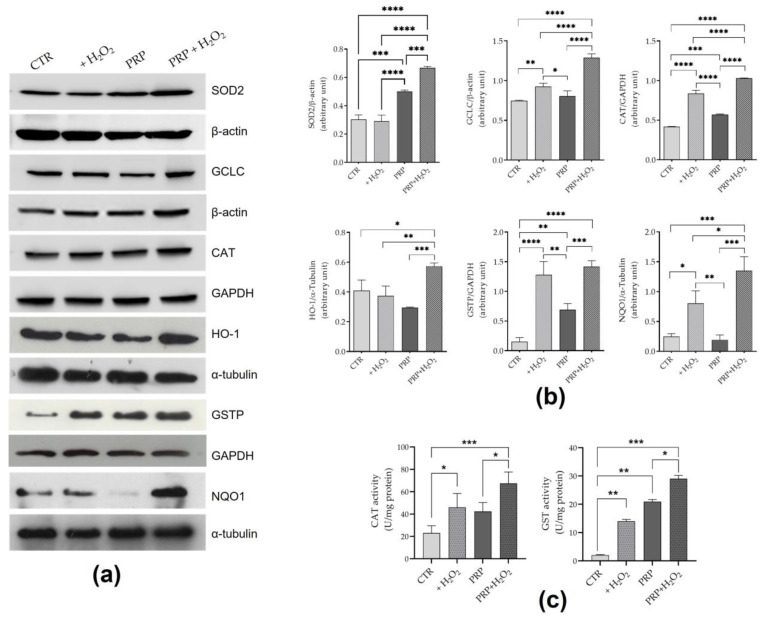
Effect of PRP of levels and activity of antioxidant enzymes. Cells treated with or without H_2_O_2_ (1 mM) for 24 h in medium with FBS (10%) (CTR) or PRP (10%) were lysed to analyze the levels of antioxidant enzymes, including GSTP, CAT, SOD2, GCLC, NQO1, and HO-1. β-actin, α-tubulin, and GAPDH are used as housekeeping protein. Representative Western blotting images (**a**) and correspondent relative bar graphs (**b**) of data obtained from three independent experiments are shown. The data are expressed as mean ± SD. **** *p* < 0.0001, *** *p* < 0.001, ** *p* < 0.01, * *p* < 0.05. (**c**) Enzyme activities of GST and CAT measured on cellular lysates obtained from tenocytes exposed for 24 h to (1 mM) H_2_O_2_ in medium supplemented with 10% FBS or 10% PRP. The data expressed as mean ± SD are derived from three different experiments. *** *p* < 0.001; ** *p* < 0.01; * *p* < 0.05.

To verify if the nuclear migration of Nrf2 induced by PRP can result in the modulation of tenocytes antioxidant response, the levels of antioxidant/phase II detoxifying enzymes were investigated. The results (Figure 5a) showed that in tenocytes exposed to H_2_O_2_, the levels of catalase (CAT) and glutathione S-transferase (GSTP) significantly increased both in the presence and in the absence of PRP. The supplementation of the medium with PRP also induced a significant increase in both proteins. Furthermore, tenocytes were able to respond to the oxidative insult by significantly increasing the levels of NAD(P)H oxidoreductase quinone-1 (NQO1) and glutamate cysteine ligase catalytic subunit (GCLC) even if to a lesser extent than when PRP is present in the medium together with H_2_O_2_. PRP up-regulates superoxide dismutase 2 (SOD2) expression both in the presence and in the absence of H_2_O_2_, while inducing a statistically significant increase in heme oxygenase 1 (HO-1) levels only in tenocytes in co-presence of H_2_O_2_. In the presence of H_2_O_2_, the enzymatic activities of Glutathione Transferase (GST) and CAT were significantly increased compared to the control (Figure 5b). This effect was enhanced by the presence of PRP in the medium.

## 3. Discussion

OS plays a key role in the pathogenesis of tendinopathies and in the failure of tendon healing [12,31,32]. Tendons are continuously exposed to ROS during normal and intense physical exercise. However, an excess of these molecules, combined with other factors such as age, lifestyle and genetics, can cause inflammation, which can jeopardize proper tendon function and healing.

In this study, OS condition was simulated, in vitro, by exposing equine tenocyte cells to H_2_O_2_ and the cytoprotective and potential antioxidant effects of PRP, and the activation of Nrf2 signaling pathways was evaluated. Although the PRP has been used to treat both traumatic and degenerative tendon disorders reaping, the molecular mechanisms underlying the effects of this autologous therapy have not yet been fully understood. Platelets (PLT) secrete many GFs that play a major role in modulating cell pathways that promote tissue repair and remodeling. Moreover, it has also been demonstrated that PRP may promote tissue/cell homeostasis by counteracting the effects of OS in vivo [28,33,34] and in vitro [22,35,36,37]. To date, there are few, inconsistent data on the antioxidant role of PRP on tenocytes and tendon healing [22,38,39] so far.

After the isolation, the tenocytes cell culture was characterized by immunofluorescence by means of different antibodies, which confirmed the tenocyte nature of the cells. Actually, cultured cells showed strong immunolabeling with anti-vimentin antibody, as expected, and also a strong positivity for anti-tenomodulin antibody, a highly tendon-specific protein involved in tenocyte proliferation and tendon maturation [40]. Cultured cells showed also moderate and variable cytoplasmic positivity for collagen I, similarly to that reported for human tenocyte cell culture [41]. Interestingly, cell cultures showed positivity for SOX-9, as already described in both human and equine tenocytes [42,43].

As expected, 24 h of exposure to H_2_O_2_ reduced the tenocyte cell viability, while the co-exposure of PRP was able to neutralize or inhibit H_2_O_2_ cytotoxicity. Similar effects of PRP have been observed in gingival fibroblasts [35] and spermatozoa [37] exposed to H_2_O_2_ and PRP. Moreover, PRP proved to be capable of protecting different types of cells from various adverse conditions induced by chemical and environmental toxicants [44,45,46] that can trigger OS. Contrastingly, to the best of the authors’ knowledge, limited data exist on the effectiveness of PRP in reducing the oxidative damage of proteins induced by contraction-induced ROS generation [28,45], which can be one of the first targets of ROS raise occurring in tendons after physical exercise and overuse injuries [47].

The results of this study show that PRP reduces the protein oxidative damage induced by pro-oxidative factors. In particular, we measured the levels of carbonylated proteins and 4-HNE-protein adducts, which are both direct markers of irreversible protein oxidation. PRP reduces the protein carbonylation induced by H_2_O_2_ treatments. Indeed, elevated protein carbonyl levels were detected in tenocytes treated with H_2_O_2_ alone compared to control cells and cells exposed to H_2_O_2_ in medium supplemented with PRP_._ Moreover, our results evidenced that tenocytes exposed to PRP alone have similar protein carbonylation levels to control cells, in contrast to the previous findings reported by Hudgens and co-workers [39]. A similar trend was observed when 4-HNE protein adducts were assayed. Furthermore, since 4-HNE is an end product of lipid peroxidation [48] that easily reacts with macromolecules, our results indirectly suggested a decrease in lipid oxidative damage in the presence of PRP, as previously observed in various in vivo and in vitro models exposed to OS [28,33,45,49]. The capability of PRP to protect proteins from irreversible oxidation and exposure to H_2_O_2_-induced cell death is clearly due to its antioxidant proprieties [50]. Since antioxidant cell response is mediated primarily by Nrf2, which also plays an important role in various regenerative processes, we analyzed its translocation into the nucleus and the levels and/or activities of some antioxidants and phase II detoxifying enzymes which are products of antioxidant response elements (ARE)-driven genes [51].

Nrf2 signaling activation in cells by different agents could be a useful strategy for inhibiting oxidative injury caused by H_2_O_2_ or other oxidative stimuli [52,53,54]. However, H_2_O_2_ can serve as signaling molecule involved in modulating redox-sensitive signal transduction and can stimulate the antioxidant cellular response. In this study, we found that PRP treatment in the presence or in absence of oxidative stimuli is able to reduce Nrf2 cytosolic levels and that tenocytes co-exposed to H_2_O_2_ and PRP showed a significant increase in nuclear Nrf2. The translocation of Nrf2 induced by PRP may lead to the activation of ARE regions in tenocytes, as reported by Tohidnezhad et al. [22] and co-workers using a different approach to ours. Our results demonstrated that PRP can induce changes in the levels of some antioxidant enzymes modulated by the Nrf2/ARE pathway. In particular, increased levels of all tested enzymes, namely HO-1, GSTP1, CAT, NQO1, and GCLC, were found in cells co-exposed to pro-oxidant conditions and PRP. Moreover, increases in enzyme activities of CAT and GST activity were also observed. H_2_O_2_ increased the levels of some tested enzymes, demonstrating that tenocytes activate the antioxidant defense when exposed to pro-oxidative conditions [55]; however, this response does not effectively protect cells from cell death or from lipid and protein oxidative damage. PRP may protect tenocytes by strengthening the cellular antioxidant capacity, and these effects may be due to the known GFs released by the PLT granule [56] or other antioxidants contained in plasma that act synergically [57]. The reduction of OS induced by PRP may be essential for promoting and accelerating tendon healing [18]. Notably, the increased SOD2 levels that were only observed in the presence of PRP may help to restore tendon homeostasis, as SOD2 gene expression and SOD activity has been described in a murine model of tendinopathy [58]. Moreover, SOD2 is a mitochondrial isoform that has been associated with mitochondrial dysfunction linked to development of tendinopathy [59]. By increasing antioxidants levels in tendons, PRP can also reduce tendon adhesion (fibrotic tissue accumulation between injured tendon and the surrounding tissue), which impairs the tendon healing process; in fact, low glutathione levels and SOD activity as well as, high lipid peroxidation markers have been reported in adhesion formation during tendon repair in different models [10,23].

## 4. Materials and Methods

### 4.1. Material

All cell culture reagents and the BCA protein assay kit were purchased from Euroclone S.p.A. (Milan, Italy). Cell lysis buffer and Nrf2 rabbit mAb, HO-1 rabbit mAb, β-actin mouse mAb, GAPDH rabbit mAb, α-tubulin rabbit mAb, NQO1 mouse mAb, SOD2 rabbit mAb, anti-rabbit immunoglobulin G (IgG), horseradish peroxidase (HRP)-linked antibody, and anti-mouse IgG HRP-linked antibody were purchased from Cell Signaling Technology (Waltham, MA, USA). Collagen 1 mouse mAb, tenomodulin mouse mAb, transcription factor SOX9 rabbit pAb, anti-goat pAb Lamin B, and anti-goat HRP-linked were obtained from Santa Cruz Biotechnology (Santa Cruz, CA, USA). Vimentin rabbit pAb and Brilliant II SYBER Green qPCR Master Mix was bought from Dako Agilent (Santa Clara, CA, USA). Protease inhibitor cocktail, Pierce ECL Plus system, anti-rabbit monoclonal antibody DNP, Bovine Serum Albumin (BSA), 2,4-dinitrophenylhydrazine (DNPH), Ponceau, Comassie Brilliant Blue G-250, Triton X-100, dithiothreitol (DTT), 3-(4,5-dimethylthiazol-2-yl)-2,5-diphenyltetrazolium bromide (MTT), ethylene diamino tetra-acetic acid (EDTA), and phenylmethylsulfonyl fluoride (PMSF) were obtained from Sigma-Aldrich (St. Louis, MI, USA). A CAT activity assay kit was purchased from Elabscience (Houston, TX, USA). Anti-rabbit CAT antibody, Alexa Fluor 488/647, and DAPI were purchased from Abcam (Cambridge, UK). Anti-rabbit polyclonal antibody GCLC subunit was purchased from BIOSS (Woburn, MA, USA). Anti-mouse monoclonal antibody GSTP was purchased from BD (San Jose, CA, USA). Polyvinylidene difluoride (PVDF) membrane was purchased from Millipore (Bedford, MA, USA). Monoclonal 4-HNE antibody was acquired from R&D Systems; ECL Clarity was purchased from BioRad (Hercules, CA, USA).

### 4.2. Animals and Sample Collection

This study was performed using tenocytes isolated from SDFT. The SDFTs were obtained from 7 Arabian horses (3–10 years of age) slaughtered at local abattoirs. The tendons were excised and stored in sterile saline until use within 2–4 h after horse death. Tissues were washed three times in Dulbecco’s phosphate-buffered saline (PBS) without Ca^2+^ and Mg^2+,^ containing penicillin (100 U/mL), streptomycin (100 mg/mL), and amphotericin B (250 μg/mL). The animal procedures were approved by the Ethical Committee of the University of Perugia (prot.n. 2014-014). PRP was obtained from whole plasma derived from healthy horses according to the guidelines of the Animal Care and Use Committee of the University of Perugia.

### 4.3. Isolation of Primary Equine Tenocytes

Tendon cells were obtained from internal sections of the tendon. Using sterile scalpels, the pieces of tendon were minced and placed in the culture medium consisting of Dulbecco’s Modified Eagle Medium (DMEM) supplemented with 10% fetal bovine serum (FBS), 100 U/mL penicillin, 100 μg/mL streptomycin. After 10 days of culture in a humidified 5% CO_2_ atmosphere at 37 °C, the tissue pieces were discarded and cells that spontaneously migrated were cultivated in the same conditions as described above upon reaching confluency. The medium was changed every 48 h. Cells at 2–3 passages of subcultures were used for all experiments.

### 4.4. PRP Preparation

PRP was prepared from whole blood using the double centrifuge method reported by Bianchini et al. [60]. Blood collected from the horse jugular vein in acid citrate-dextrose (ACD) solution Vacutainer^®^ underwent two centrifugation steps (I° 200× *g* for 20 min at 25 °C; II° 1800× *g* for 10 min at 25 °C). PLT pellet was then resuspended in a volume of platelet-poor plasma to obtain a final platelet concentration of 2 × 10^6^ PLT/μL [61]; PLT, counts were determined with a hemacytometer (EosBIO, Cervarese Santa Croce, Italy).

### 4.5. Immunofluorescent Imaging

On the second passage of subculture—the same passage which was used for all experiments—tenocytes were grown on glass coverslips for immunofluorescence phenotypization. Cells were washed with PBS and fixed with 4% paraformaldehyde (PFA) in PBS for 20 min at room temperature and then washed 3 times with PBS. After permeabilization with 0.1% Triton X-100 in PBS for 15 min and blocking with 2% BSA in PBS for 60 min, the cells were incubated overnight at 4 °C with primary antibodies against collagen I (1:200), tenomodulin (1:100), vimentin (1:100) and SOX9 (1:100). Afterwards, secondary antibodies raised against mouse IgG and rabbit IgG conjugated with a fluorochrome (Alexa Fluor 488 and Alexa Fluor 647) were incubated for one hour at room temperature. Finally, glass coverslips with immunolabeled cells were mounted with an aqueous mounting medium containing DAPI. The slides were then evaluated using a fluorescent microscope Olympus BX51, equipped with the camera Nikon mod.DS-Qi2Mc. NIS-ELEMENTS D software (version 5.10) was used for image acquisition.

### 4.6. Analysis of Cell Viability

The effect of H_2_O_2_ and PRP on tenocytes viability was evaluated using the MTT colorimetric assay [62]. Briefly, cells were seeded at a density of 1 × 10^4^ cells/well on 96-well plates. After 1 day of culture, media were removed and fresh media (100 µL), supplemented with 10% FBS or 10% PRP, containing increasing concentrations of H_2_O_2_ (0.1, 0.5, 1, 2 mM) of an original suspension equal to 2 × 10^6^ PLT/μL were added (in a humidified 5% CO_2_ atmosphere at 37 °C, 24 h). Cells maintained in medium supplemented with 10% FBS or 10% PRP without H_2_O_2_ were assumed as controls. After treatment, the cells were incubated with complete medium containing MTT (0.5 mg/mL). After 2 h of incubation, the media were removed and 200 µL of DMSO was added to each well to dissolve the formed formazan crystals. The absorbance was measured using a plate spectrophotometer (Infinite^®^ 200 Pro-Tecan, Mennedorf, Switzerland) at 570 nm. The percentage of viable cells relative to the control was then calculated, assuming the absorbance of the controls was 100% (absorbance of treated wells/absorbance of control wells × 100).

### 4.7. Protein Extraction and Immunoblotting

To extract total proteins, cells were detached using Trypsin-EDTA solution, washed, and resuspended in 100 μL of ice-cold Cell Lysis Buffer with 20 μL/mL protease inhibitor cocktail, and lysed according to the manufacturer’s instructions. The cellular proteins were collected, and a BCA protein assay kit was used to measure total protein concentrations; BSA was used as an external standard.

Nuclear and cytosolic protein extractions were performed to evaluate the Nrf2 cytosolic and nuclear content. Briefly, cells were resuspended at 4 °C in Buffer 1 containing 10 mM HEPES (pH 7.9), 1.5 mM MgCl_2_, 10 mM KCl, 0.5 mM DTT, and 0.2 mM PMSF, allowed to swell on ice for 10 min, and then vortexed for 10 s. Samples were centrifuged at 10.000× *g* for 2 min, and the supernatant containing the cytosolic fraction was stored at −80 °C. The pellet was resuspended in cold Buffer 2 containing 20 mM HEPES (pH 7.9), 25% glycerol, 420 mM NaCl, 1.5 mM MgCl_2_, 0.2 mM EDTA, 0.5 mM DTT, 0.2 mM PMSF, 2.5 μg/mL leupeptin, and 2.5 mg/mL aprotinin and incubated on ice for 20 min for high salt extraction. Cellular debris was removed via centrifugation at 13,000× *g* for 10 min at 4 °C, and the supernatant fraction containing nuclear protein extract was stored at −80 °C. Proteins were measured using the BCA protein assay kit.

For immunoblotting, proteins (20–25 μg) were resolved via 10 to 12% sodium dodecyl sulphate polyacrylamide gel electrophoresis (SDS–PAGE) and then transferred to PVDF membrane for immunoblot analysis. The blocked membranes were incubated overnight at 4 °C with the primary antibodies anti Nrf2 (1:1000), HO-1 (1:1000), NQO1 (1:1000), SOD2 (1:1000), GCLC (1:000), CAT (1:1000), GSTP (1:1000), β-actin (1:1000), GAPDH (1:1000) α-tubulin (1:1000), Lamin B (1:1000), 4-HNE (1:1000). Membranes were washed and then incubated with the secondary antibodies: anti-rabbit immunoglobulin G (IgG), horseradish peroxidase (HRP)-linked antibody (1:3000), anti-goat HRP-linked antibody (1:10,000) or anti-mouse IgG, HRP-linked antibody (1:5000) for 1 h at room temperature. After washing with tris-buffered saline, tween 0.1% TBST, immunoreactive signals were detected, as evidenced by the chemiluminescence reaction detected using the Pierce ECL Plus system or ECL Clarity. The immunoreactive proteins were highlighted via a chemiluminescence reaction. Images were acquired using a GS-800™ Calibrated Densitometer (Bio-Rad, Hercules, CA, USA).

### 4.8. Evaluation of Protein Oxidation

In order to evaluate the oxidative damage of proteins, the levels of both protein-4HNE and carbonylated proteins were evaluated. The determination of carbonylated proteins was performed using OxyBlot in accordance with Colombo et al. [63], via direct protein labeling with DNPH. Next, 30 μg of DPN-proteins was subjected to SDS-PAGE and immunoblotting, as described above. After overnight incubation with anti-DNP (1:10,000) and detection of immunoreactive DNP-labeled proteins, the PVDF membranes were then stained with Comassie Brilliant Blue G-250 to define the total protein content of each lane and normalized data. The film and filter images were acquired using a GS-800 imaging systems scanner. Densitometric analysis was performed using Quantity One (Biorad, Hercules, CA, USA).

The analyses of protein-4HNE were performed by immunoblotting as described above. The immunoreactive 4HNE-proteins were evidenced by chemiluminescence reaction using a ChemiDoc Imaging System (Biorad, Hercules, CA, USA) and the nitrocellulose membranes were then stained with Ponceau, in order to define the total protein content of each lane and normalized data. The film and filter images were acquired using a GS-800 imaging systems scanner. Densitometric analysis was performed using Quantity One (Biorad, Hercules, CA, USA).

### 4.9. Enzyme Activities

The specific activity of the GST in tenocytes was measured according to Zhang et al. [64], using 5 mM GSH and 0.5 mM CDNB as the second substrate in 0.1 M potassium phosphate buffer pH 6.5 at room temperature. The changes in absorbance at 340 nm were monitored for 5 min with the UV/visible spectrophotometer (Thermo-Fisher Scientific, Waltham, MA, USA). The molar extinction coefficient used for CDNB conjugation was 9.6 mM^−1^ cm^−1^. Enzymatic activities were calculated after correction for the non-enzymatic reaction.

The CAT activity in horse tenocytes was measured using the CAT activity assay kit according to the manufacturer’s instructions (Elabscience, E-BC-K031-S). The UV/visible spectrophotometer used to measure CAT activity was a JascoV-550 ETC 505S (Portland, OR, USA).

### 4.10. Statistical Analysis

Statistical analysis was performed using analysis of variance (ANOVA) with Tukey correction as a post hoc test for multiple comparisons. *p*-values below 0.05 were considered statistically significant. All data are expressed as mean ± SD (standard deviation).

## 5. Conclusions

The data obtained from this study revealed that PRP protects tenocytes from the adverse effects of pro-oxidant agents, which are known to be etiopathological factors of tendinopathies and one of the main causes of healing failure. In particular, PRP appears to enhance the antioxidant capacity of tenocytes via Nrf2 signaling, thus alleviating the effects of oxidative stress and reducing or preventing molecular oxidative damage.

Our findings provide valuable insights into the molecular mechanisms underlying the natural tendon healing properties of PRP. The results could suggest that PRP may activate various cellular pathways that restore tenocyte homeostasis to promote tendon regeneration and repair following tendon injuries, which are well known for their poor healing potential and high recurrence rates. In conclusion, this study provides new insights into the management of equine tendinopathy and PRP therapy. Finally, as the SDFT is one of the best animal models of the human Achilles tendon, the findings presented here could have putative translational value for therapeutic strategies of human tendon disorders.

## Data Availability

The data presented in this study are available on request from the corresponding author.
